# Leptin- and Leptin Receptor-Deficient Rodent Models: Relevance for Human Type 2 Diabetes

**DOI:** 10.2174/1573399810666140508121012

**Published:** 2014-03

**Authors:** Bingxuan Wang, Charukeshi Chandrasekera P., John J. Pippin

**Affiliations:** Physicians Committee for Responsible Medicine, 5100 Wisconsin Avenue NW, Washington, DC 20016, USA

**Keywords:** Diabetes, leptin/leptin receptor mutations, obesity, rodent models, translational barrier.

## Abstract

Among the most widely used animal models in obesity-induced type 2 diabetes mellitus (T2DM) research are the congenital leptin- and leptin receptor-deficient rodent models. These include the leptin-deficient ob/ob mice and the leptin receptor-deficient db/db mice, Zucker fatty rats, Zucker diabetic fatty rats, SHR/N-cp rats, and JCR:LA-cp rats. After decades of mechanistic and therapeutic research schemes with these animal models, many species differences have been uncovered, but researchers continue to overlook these differences, leading to untranslatable research. The purpose of this review is to analyze and comprehensively recapitulate the most common leptin/leptin receptor-based animal models with respect to their relevance and translatability to human T2DM. Our analysis revealed that, although these rodents develop obesity due to hyperphagia caused by abnormal leptin/leptin receptor signaling with the subsequent appearance of T2DM-like manifestations, these are in fact secondary to genetic mutations that do not reflect disease etiology in humans, for whom leptin or leptin receptor deficiency is not an important contributor to T2DM. A detailed comparison of the roles of genetic susceptibility, obesity, hyperglycemia, hyperinsulinemia, insulin resistance, and diabetic complications as well as leptin expression, signaling, and other factors that confound translation are presented here. There are substantial differences between these animal models and human T2DM that limit reliable, reproducible, and translatable insight into human T2DM. Therefore, it is imperative that researchers recognize and acknowledge the limitations of the leptin/leptin receptor-based rodent models and invest in research methods that would be directly and reliably applicable to humans in order to advance T2DM management.

## INTRODUCTION

Type 2 diabetes mellitus (T2DM), or non-insulin-dependent diabetes mellitus, is a disease of chronic hyperglycemia that leads to severe and sometimes fatal complications. Over 26 million Americans currently have diabetes and an additional 79 million are considered pre-diabetic; 90-95% of diagnosed cases of diabetes are T2DM. In addition to costing hundreds of billions of dollars annually, T2DM is one of the leading causes of death, the leading cause of kidney failure, and a major cause of heart disease in the United States [1, 2]. Globally, the incidence of T2DM is increasing rapidly, and the World Health Organization predicts that by 2030 the prevalence of T2DM will double to 350 million worldwide [3]. As a result, the needs for means to prevent, diagnose, and treat T2DM and its complications are ever-increasing. 

Over the past several decades, considerable resources have been devoted to T2DM research using animal models, and the merits and limitations of various animal models have been reviewed elsewhere [4-12]. The species commonly used include cats, dogs, nonhuman primates, and especially rodents. Depending on the method(s) used to induce diabetes, these animal models can be broadly classified into spontaneous/congenital, diet-induced, chemical-induced, surgical, and transgenic diabetic models [12]. Among the most frequently used are the spontaneously diabetic rodent models deficient in leptin or leptin receptors. These models include leptin deficient ob/ob mouse, leptin receptor deficient db/db mouse, Zucker fatty rat, Zucker diabetic fatty (ZDF) rat, spontaneously hypertensive/NIH corpulent (SHR/N-cp) rat, and JCR:LA-cp rat (note that this is not a comprehensive list of all leptin or leptin receptor deficient animals since not all such models are widely used for T2DM research). Due to single-gene mutations that lead to the lack of action by the satiety factor leptin or its cognate receptor, these rodents spontaneously develop severe hyperphagia leading to obesity and manifest some T2DM-like characteristics. Of these models, the ob/ob mouse, db/db mouse, Zucker fatty rat, and ZDF rat represent some of the earliest animal models developed and remain among the most widely used models for T2DM research today generating over 4000 publications on PubMed over the last decade alone. Other corpulent (designated cp) rat models emerged later and are mainly used to study diabetic cardiovascular complications. Despite the extensive use of these rodent models, many mechanistic details of human T2DM remain poorly understood and treatment options for humans are limited and largely unsatisfactory. The purpose of this review is to analyze the experimental evidence and evaluate the relevance of leptin and leptin receptor-deficient rodent models for human T2DM. 

### T2DM IN HUMANS

The natural history of T2DM in humans leads from insulin resistance to compensatory hyperinsulinemia, pancreatic β-cell dysfunction, impaired glucose tolerance, and finally T2DM characterized by overt hyperglycemia [13]. The development of T2DM is typically slow in humans, and individuals may be asymptomatic for many years. Diverse factors contribute to insulin resistance and β-cell dysfunction, and therefore human T2DM is termed “multifactorial,” in contrast to monogenic animal models of T2DM. Both genetic and acquired factors influence T2DM susceptibility in humans. Human T2DM is associated with a number of risk factors which include obesity, advancing age, genetic predisposition (family history of diabetes and ethnic background), previous history of gestational diabetes, suboptimal intrauterine environment, low birth weight, and lifestyle factors such as consumption of high caloric diets, stress, and physical inactivity [14]. T2DM-induced complications can be categorized into macrovascular and microvascular conditions and account for the vast majority of T2DM-related morbidity and mortality [15]. 

The importance of genetic predisposition to T2DM has been demonstrated in twin studies [16]. Several approaches have been used for the discovery of candidate genes: linkage analysis, candidate gene association studies, and genome-wide association studies [17]. To date, polymorphisms of more than three dozen genes have been reported to be linked to T2DM, but leptin and leptin receptor genes are not among them [14, 17-19]. Moreover, since T2DM is a multifactorial disease and multiple genes are involved, each sequence variation of one gene may contribute minimally or not at all to increased T2DM susceptibility. Thus, it is crucial to evaluate various aspects of the leptin- and leptin receptor-based animal models in light of their ability to appropriately mimic aspects of T2DM in order to ensure humans can benefit from such research findings.

### LEPTIN/LEPTIN RECEPTOR-DEFICIENT RODENT MODELS IN RESEARCH AND DRUG DEVELOPMENT

In basic research, ob/ob mice, db/db mice, Zucker fatty rats and ZDF rats have been extensively used to study the pathogenesis of T2DM, obesity, leptin signaling, and the interactions among the three. All these animal models are obese and insulin resistant with dyslipidemia and virtually all mechanistic aspects of T2DM have been examined in these animals. In drug discovery and testing, the db/db mouse is the most popular animal model used by pharmaceutical companies to test glucose lowering agents, insulin sensitizers, insulin secretagogues, and anti-obesity agents, although ob/ob mice, Zucker fatty rats and ZDF rats are also widely used [20]. The corpulent rats are less used, possibly due to their late emergence. A major advantage of spontaneous/congenital diabetic animal models is that researchers do not need to use time-consuming feeding schemes or invasive procedures that risk side effects to induce the diabetic symptoms. However, when the diabetic manifestations in some of these models do not develop in females (as detailed later in the text) or are not as apparent as desired, dietary manipulations as well as transgenic efforts have been combined to “improve” the models. Taken together, these models have been widely used for various purposes, and the following sections will address in detail the molecular basis and the pathophysiological manifestations of these models as well as how they compare with human T2DM disease state. 

### MOLECULAR BASIS FOR LEPTIN AND LEPTIN RECEPTOR DEFICIENCY

**Fig. (1):**
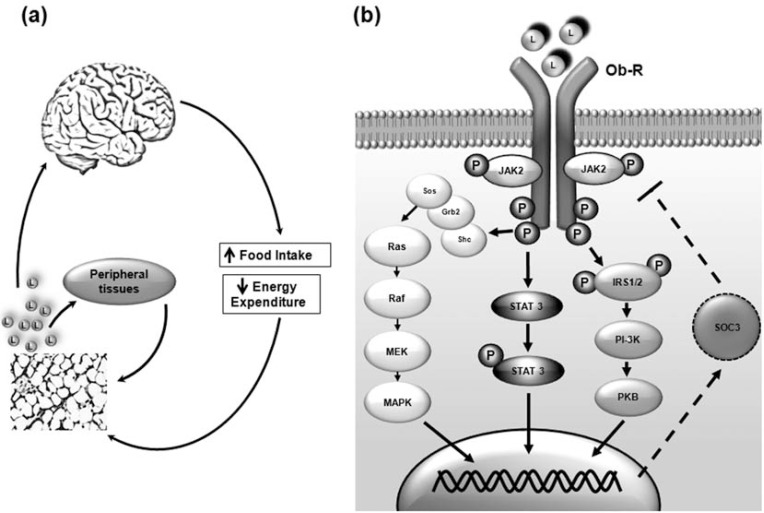
Leptin Signal Transduction. a) Peripheral action of leptin: leptin is produced by adipose tissue and circulates to the brain, primarily the hypothalamus where it modulates food intake and energy expenditure via several signal transduction pathways. Leptin also binds to various peripheral tissues and regulates leptin production via feedback modulation. b) Leptin-mediated signaling: binding of leptin to its cognate receptor (Ob-R) activates three key signaling cascades in the hypothalamus (JAK/STAT, MAPK, and PI3K pathways), which result in the transactivation of various signaling molecules and subsequent gene transcription. Leptin (L), Janus kinase/signal transducer and activator of transcription (JAK/STAT), mitogen-activated protein kinase (MAPK), phosphatidylinositol 3-kinase (PI3K), protein kinase B (PKB); growth receptor-bound-2 (Grb2), insulin receptor substrate (IRS), SH2-domain containing protein tyrosine phosphatase (SH2), suppressor of cytokine signalling-3 (SOC3). refers to activation; → refers to inhibition.

Leptin is a hormone produced primarily by the mature adipocytes in white adipose tissue and to a lesser extent in other tissues such as brown adipose tissue, skeletal muscle, placenta, ovaries, bone marrow, and stomach. Circulating leptin is taken up into the brain where it mainly acts to regulate food intake, appetitive behaviors, and energy expenditure (Fig. **1A**). Besides its role in satiety, leptin has been implicated in various endocrinological and physiological processes including the regulation of energy homeostasis, thermogenesis, reproduction, hematopoiesis, skeletal growth, neuroprotection, and oncogenesis [21, 22]. Due to the multifaceted role of leptin, spontaneous mutations have led to leptin signaling abnormalities, which were among the earliest factors discovered to cause diabetes-like symptoms in rodents. 

Leptin peptide is encoded by a single gene where full-length leptin is translated as a 167 amino acid peptide while circulating leptin is only 147 amino acids following signal sequence removal. Leptin exerts its physiological actions *via* the cognate leptin receptor, Ob-R. Alternative splicing of the mammalian Ob-R yields six isoforms of varying intracellular domain lengths (Ob-Ra→f). The long form Ob-Rb is mainly expressed in the hypothalamus and is believed to be responsible for transducing central actions by leptin. The short intracellular forms of Ob-R are less understood, but they are widely expressed in various peripheral tissues [22]. Upon leptin binding, Ob-R homodimerizes and couples to the JAK/STAT pathway, and subsequent tyrosine phosphorylation initiates several other downstream signaling cascades capable of exerting various physiological consequences (Fig. **1B**). 

Several spontaneously occurring loss-of-function mutations in leptin and leptin receptors result in numerous physiological consequences in rodents and humans. The general characteristics of the four most characterized rodent mutations that affect the leptin signaling system are described below. Although they all result in leptin or leptin receptor deficiency, the manifestations of these mutations relevant to T2DM can be quite different. More detail regarding the mechanism of T2DM pathology in rodent models and humans is provided in following sections. 

#### The ob Mutation

Diabetes was identified in ob/ob mice in the late 1940s and later linked to a single autosomal recessive mutation on the obese gene (leptin encoding gene on chromosome 6, *Lep^ob^*). A nonsense mutation (C to T) in codon 105 changes an Arg residue to a stop codon causing premature truncation, rendering the translated protein biologically inactive. As a result, although there are high levels of leptin mRNA in adipocytes, the animals completely lack functional leptin [23, 24]. 

#### The db Mutation

The diabetic characteristics of db/db mice also derive from a single autosomal recessive mutation. This is a Gly to Thr mutation in the leptin receptor gene on chromosome 4 (Lepr^db^), resulting in abnormal mRNA splicing and the subsequent production of a nonfunctional Ob-Rb protein. As mentioned above, Ob-Rb encodes the only protein that has a longer cytoplasmic domain and is highly expressed in particular sites within the central nervous system. The mutation in db/db mice leads to the functional replacement of Ob-Rb by Ob-Ra [25-27]. The defective leptin receptor leads to the over-production of extracellular leptin, but lack of intracellular leptin action through Ob-Rb. 

#### The fa Mutation

Long and short forms of Ob-R were also identified in rats. In the Zucker fatty (fa) rats, a missense A to C mutation in the Lepr gene on chromosome 5 (Lepr^fa^) causes a Gln to Pro change in all the identified isoforms of Ob-R protein. Although mRNA transcripts and proteins of all the isoforms are still produced in this strain, they are nonfunctional due to the mutation [28]. A sub-strain of Zucker fatty rats, known as the ZDF rats, is selectively inbred for hyperglycemia. They carry an autosomal recessive defect in β-cell transcription that is inherited independently from the Lepr mutation. The gene responsible for the defect has not been identified, but this defect in itself is not sufficient to cause diabetes – only when combined with Lepr mutation can it lead to hyperglycemia [29]. The fa/fa genotype has also been transferred to other rat strains giving rise to other T2DM models such as the Wistar diabetic fatty (WDF/Ta-fa) rat and WKY fatty rat [30]. Compared to Zucker rats, these models are relatively new and less extensively studied. Consequently, they are not discussed in detail in this article.

#### The cp Mutation

The corpulent (cp) phenotype resulting from an autosomal recessive mutation on the Lepr gene was first recognized in the obese spontaneous hypertensive rats (SHR) [31]. This mutation is a Thr2349Ala transversion, leading to a premature stop codon in the extracellular domain of leptin receptor protein just before the transmembrane domain. As a result, all the leptin receptor isoforms produced are nonfunctional [32], and these rats are leptin-resistant in the face of high levels of circulating leptin [31]. Since the discovery of cp/cp characteristics in Koletsky rats, several sub-strains have been bred from this strain and used as T2DM models, including the SHR/N-cp rat, the SHHF/Mcc-cp rat and the JCR:LA-cp rat. Since these corpulent rats are phenotypically almost identical, only the SHR/N-cp rat and the JCR:LA-cp rat will be discussed in this paper. All the rats homozygous for the cp gene lack functional leptin receptors. 

#### Human Leptin/Leptin Receptors

In humans, plasma leptin levels are positively correlated with body mass index (BMI) and body fat. Leptin expression and leptin secretion by adipose tissue is thought to be regulated by nutritional status (fasting and feeding), insulin, steroids (glucocorticoids and sex steroids), and perhaps other hormones as well as β-adrenergic action on adipocytes [33]. Since obesity is a risk factor for T2DM, the relationship between leptin and T2DM is being extensively studied. Epidemiological studies have suggested that leptin regulates total body sensitivity to insulin and triglyceride levels in leptin-deficient individuals [34], and there is a negative relationship between insulin resistance and cerebrospinal fluid leptin concentrations [35]. However, association studies trying to link leptin polymorphisms and obesity in humans have produced conflicting results [36-38]. Both obese and T2DM subjects are leptin-resistant, but studies are not in agreement with regard to plasma leptin levels in T2DM patients. They were reported to be higher, lower, or similar to BMI-matched non-diabetic controls [39, 40]. In addition, humans with inactivating mutations in leptin or its receptor have less severe endocrine disturbances, compared with leptin-deficient rodents, suggesting that leptin may be less critical in the regulation of energy expenditure in humans [41]. Recombinant leptin therapy is effective in rare cases of congenital leptin deficiency. On the other hand, it has limited or no success in treating obesity and T2DM [42]. 

### MANIFESTATIONS OF DIABETES AND UNDERLYING MECHANISMS 

Obesity is the primary phenotypic manifestation observed in the various leptin- and leptin receptor-deficient rodents, but they also display some T2DM-like characteristics such as hyperglycemia, glucose intolerance, and elevated plasma insulin. The comparative analysis of diabetic manifestations between these rodent models and humans is summarized in (Table **1**). In the following sections, we analyze T2DM in two mouse models (ob/ob and db/db), four rat models (Zucker fatty rat, ZDF, SHR/N-cp, and JCR:LA-cp), and humans, focusing primarily on six key manifestations of T2DM (obesity, hyperglycemia, hyperinsulinemia, insulin resistance, macro- and microvascular complications) and other relevant features.

### Obesity 

#### Mouse Models

The most common and striking feature of all six leptin-related rodent models is their early and severe obesity. Homozygous ob/ob and db/db mice have severe, rapid, spontaneous and early-onset obesity that is first recognizable at about 4 weeks of age. Their body weight can reach three times the normal weight of wild-type controls [43], and these changes are visible at the cellular level, as seen by increased adipocyte number and size. In the human population, a large majority of T2DM patients are overweight or obese, and obesity is the number one risk factor for T2DM. However, obesity can occur at any point throughout life, with increasing likelihood with advancing age, unlike in these animal models displaying only early-onset obesity. Moreover, the degree of obesity in human T2DM patients is variable, and is usually not as severe as in these rodents this early in life, which can affect many aspects of development.

Obesity in these models is primarily due to uncontrolled appetite, hyperphagia, and reduced energy expenditure, which are directly caused by leptin signaling abnormalities [44, 45]. It is notable that administration of leptin corrects many diabetic manifestations in ob/ob mice, and the correction of hyperinsulinemia and hyperglycemia occurs before the effect on obesity [46-48], indicating that obesity plays a secondary role in the pathogenesis of diabetic manifestations in this model. As a result, studies using ob/ob mice to identify the impact of obesity on T2DM have little – if any – relevance for human patients. In humans, obesity is the result of lifestyle and multifactorial genetic inheritance [17], rather than leptin deficiency/resistance of monogenic inheritance, although obese T2DM patients often have abnormal levels of leptin, likely secondary to the development of T2DM. Interestingly, leptin levels are often elevated in obese humans and not reduced as in these rodent models. Additionally, although ob/ob and db/db mice have elevated cholesterol levels, similar to humans with T2DM, these are primarily contributed to by the HDL cholesterol fraction. In general, the reverse lipid profile (high HDL, low LDL) in mice combined with efficient lipid clearance makes them different from the human obesogenic phenotype. T2DM patients also often have dyslipidemia, characterized by elevated triglyceride and VLDL cholesterol, normal or increased LDL cholesterol and total cholesterol, and reduced HDL cholesterol. Unlike in the mice, human lipid clearance is also impaired due to decreased activity of lipoprotein lipase. [49-51].

#### Rat Models

For Zucker fatty rats and ZDF rats, obesity is observed at 3 weeks and is severe by 5 weeks of age where their weights are almost twice that of their lean heterozygous littermates. Hyperphagia is apparent during the growth period of these animals, but food consumption later returns to levels comparable to their lean littermates [52]. Obesity is associated with hyperphagia, defective non-shivering thermogenesis, increased efficiency for food utilization, and preferential deposition of energy in adipose tissue [53, 54], all triggered by leptin receptor abnormalities. As in the mouse models, these factors do not accurately correlate with human disease etiopathogenesis. Both LDL cholesterol and HDL cholesterol levels are elevated in Zucker fatty rats and ZDF rats, in addition to increased activity of lipoprotein lipase [54-56]. Aside from these general differences in the lipid profile, the lipid concentrations measured within and between studies reported by different investigators vary greatly [57], thereby making it difficult to extrapolate even within the same species harboring the same disease-causing genetic abnormality.

Obesity in cp/cp rats is detectable at 3 weeks of age and is evident in SHR/N-cp rats by 5-6 weeks [58, 59]. As with other above-mentioned models, the development of obesity is closely related to leptin receptor deficiency [60], which is not present in human T2DM subjects. Female SHR/N-cp rats are less obese than males, and both HDL cholesterol and LDL cholesterol levels are elevated in these rats [61]. However, serum triglyceride (TG) levels are substantially higher in female than male rats, and associated with increased hepatic lipogenic enzyme activities [60]. In contrast, sex differences of this nature do not exist within the human T2DM population. 

The obesity of JCR:LA-cp rats is much more extreme than observed in the Zucker rats [57]. High TG levels can be observed as early as 4 weeks of age, almost exclusively due to increased hepatic VLDL secretion rather than reduced clearance, and lipoprotein lipase activity is increased 2-4 fold in different tissues [62]. In addition, HDL cholesterol concentrations are elevated in these rats [63], and female rats develop a more severe hyperlipidemia than male rats [64]. 

With respect to gender, no significant differences in lipid profile are observed in T2DM human subjects except a higher HDL cholesterol level in women [65]. This lipid profile is different from all the above-mentioned animal models (see Table **1**). Taken together, the obesogenic phenotype present in the leptin-based rodent models of T2DM does not appropriately mimic the human etiology, natural history or pathogenesis.

### Hyperglycemia

#### Mouse Models

Hyperglycemia is the hallmark of T2DM; however, in ob/ob mice, hyperglycemia is transient and mild. It is observed at about 1 month of age and starts to decline after 3 months, and by the 7^th^ month, blood glucose levels are comparable to control mice [12, 66]. This is in marked contrast to the human condition, where hyperglycemia develops slowly and worsens over time. Hyperglycemia in the ob/ob mouse is probably caused by leptin deficiency, and its limited severity is due to sustained hyperinsulinemia, which leads to obesity as a side effect [67, 68]. In contrast, hyperglycemia develops in humans when compensatory hyperinsulinemia in response to insulin resistance is not sufficient to control blood glucose levels. 

Leptin receptor-deficient db/db mice develop hyperglycemia by 2 months of age, but not all db/db mice develop it [69]. In db/db mice at 10 weeks of age, fasting blood glucose levels can reach ~600 mg/dl in comparison to ~150 mg/dl in control mice [70]. This level of hyperglycemia is extreme compared to an average of ~200 mg/dl in human T2DM patients [71]. Although gluconeogenesis is elevated in both T2DM patients and in db/db mice, glucose clearance is reduced in human T2DM subjects, but it is elevated as much as 170% in db/db mice [72]. In addition, hepatic glycogen synthesis plays an important role in the autoregulation of blood glucose in humans [73], and liver glycogen concentration in human T2DM patients is reduced [74], whereas in db/db mice it is greater than control at all ages with a greater turnover rate [72]. 

#### Rat Models

Similar to ob/ob mice, Zucker fatty rats are not hyperglycemic [75-77]. Some male ZDF rats develop hyperglycemia (500mg/dL, compared to 200mg/dL in control) by 10 – 12 weeks of age. In addition, development of hyperglycemia appears to be gender-specific: unlike male ZDF rats, female ZDF rats do not become diabetic except through dietary modification [78, 79]. In the human population, however, hyperglycemia occurs equally in both sexes. The expression of diabetes in ZDF rats is highly dependent on the specific diet and treatment protocols. Fasting and excessive bleeding procedures can lead to a delay and inconsistent expression of diabetic manifestations [80], but such procedures are not known to exert the same effect on human T2DM. The aforementioned difference in liver glycogen is also seen between ZDF rats and human T2DM patients [81]. For this model, a substantial amount (50%) of plasma glucose after 24h fasting originates from glycogenolysis [82]. But in T2DM patients with poor glycemic control, elevated fasting blood glucose is attributed to gluconeogenesis, and their level of glycogenolysis is similar to or less than that of healthy individuals [83, 84]. 

In SHR/N-cp male rats, impaired glucose regulation appears as early as 2 months of age [85], manifesting postprandial hyperglycemia higher than 400 mg/dl [60]. However, fasting blood glucose is normal or slightly elevated [50, 86], unlike in the human disease state. Moreover, glucose tolerance in these rats improves with aging [87], in contrast to the situation in human T2DM patients. JCR:LA-cp rats do not develop hyperglycemia, due to islet β-cell insulin hypersecretion [88]. This is at variance with the human condition, where overt hyperglycemia persists following a phase of compensatory insulin hypersecretion. Fasting glucose concentrations are not increased in these rats, although marked glucose intolerance can be observed in male rats. Female rats have only moderate glucose intolerance [63]. Similar to SHR/N-cp rats, a major drawback of JCR:LA-cp rats as a model of T2DM is the lack of fasting hyperglycemia [50], a gender-independent defining feature of human T2DM. 

### Hyperinsulinemia

#### Mouse Models

Pancreatic islets of ob/ob mice are almost entirely composed of β-cells, compared to about 55% of β-cells in pancreatic islets in humans and 70-80% in wild type mice. This increase in β-cell mass may be the result of sensing increased demand for insulin [89]. These β-cells remain healthy and functional throughout life; hence the pancreas is described as “sturdy” in these mice [44]. In marked contrast, the human T2DM pancreas becomes brittle and dysfunctional with disease progression. In further contrast to the human natural history and etiology, these mice develop hyperinsulinemia within 2 weeks after birth and remain hyperinsulinemic until eight months of age, when glucose levels decrease [44]. For these reasons, ob/ob mice do not develop insulin insufficiency or diabetes and they are regarded as a good source of insulin-secreting β-cells for *ex vivo *or *in vitro* experiments. Since recombinant leptin inhibits insulin secretion in ob/ob mice [90], leptin deficiency plays a role in the development of uncontrolled insulin secretion in this model, which is irrelevant to human T2DM. The lack of leptin signaling in ob/ob mice may confound research results on β-cell functions, making them hard to translate to humans. 

Plasma insulin in db/db mice starts to rise at 10-14 days and peaks at 3 months of age at around 35 ng/ml [70, 91]. In contrast to the leptin-deficient ob/ob mice, the leptin-resistant db/db secretory functions of β-cells gradually decline around the age of 6 months [92] with severe depletion of β-cells. Within a few weeks, body weight drops rapidly, and death occurs by 10 months of age. Female mice live longer than males [92]. The severe hyperinsulinemia in these mice contrasts with the moderate condition in human T2DM patients, which may reflect different disease pathogenesis between the two species. Pancreatic islets isolated from human T2DM patients showed only ~10% β-cell destruction, which led to the belief that β-cell function loss is more critical than actual cell loss in the disease process [93]. In addition, although human pancreatic β-cells express leptin receptor Ob-Rb, the isoform that has been most extensively studied, these cells express other isoforms of leptin receptors much more abundantly [94]. Although the pancreata of db/db mice do not express functional Ob-Rb, they do have normal expressions of other Ob-Rs, but little is known about their functional significance. High circulating levels of leptin in db/db mice may therefore play a role in the development of hyperinsulinemia, and studies using these mice may potentially be confounded by this effect. 

#### Rat Models

Zucker fatty rats are similar to ob/ob mice in that they do not develop overt diabetes, and therefore are regarded as a pre-diabetes model. Hyperinsulinemia is seen at 3-4 weeks of age [95]. When these rats reach about 30 weeks of age, plasma insulin levels typically return to normal [96], which is in marked contrast to the human disease state. It is also noteworthy that these rats have reduced levels of glucagon [54], a hormone produced by pancreatic α-cells that opposes insulin action. In contrast, human T2DM patients have high levels of glucagon [97], and hyperglucagonemia is believed to play a role in the development of hyperglycemia in T2DM [98]. The deficiency of leptin and glucagon signaling may render the Zucker fatty rat an inappropriate model for the study of the complex signaling pathways in human T2DM glucose homeostasis. 

Pancreatic β-cell mass of ZDF rats increases from 6 weeks to 16 weeks of age and starts to decline thereafter through apoptosis. Plasma insulin levels increase dramatically from 6 weeks to 8 weeks old, but decline rapidly after that to levels similar to that of 6 weeks old [42, 99]. At 14 weeks, the animals become insulinopenic [100]. Glucagon-positive islet cells in young ZDF rats (9-13 weeks) are significantly higher than control rats, but this difference is insignificant by 30-34 weeks, while T2DM patients consistently have higher glucagon levels [101, 102]. In addition, in ZDF rats, leptin protects β-cells from free fatty acid-induced apoptosis [103], while in cultured human islets chronic exposure to leptin leads to β-cell apoptosis [104].

SHR/N-cp rats are hyperinsulinemic as early as 4 weeks of age [60]. The hyperinsulinemia can be exacerbated by a high-sucrose diet [105]. In 5-month old male rats, plasma insulin levels are 6 times higher than those of their lean controls [58]. The hyperinsulinemia is associated with marked hyperplasia of pancreatic β-cells, and the rats remain markedly hyperinsulinemic until death from cardiovascular complications at about 1-year old [85]. The β-cell hyperplasia, however, lacks the hyaline changes and hydropic changes seen in human islets [106]. In addition, glucagon secretion in these rats is relatively suppressed [60]. JCR:N-cp rats develop moderate hyperinsulinemia at 3 weeks of age, which rapidly progresses to a marked hyperinsulinemia much more severe than Zucker rats beyond 5 weeks old [88]. This is the result of an extreme age-dependent pancreatic islet hyperplasia which can be observed as early as 1 month of age. Both β-cells and α-cells contribute to the hyperplasia, with the former playing the major role. Glucagon level is only mildly elevated [63]. Male rats have much higher plasma insulin concentrations than female rats [64], while in the human T2DM population, females have similar or slightly higher levels of fasting plasma insulin than males [107]. Taken together, etiology and pathogenesis as well as gender presentation of hyperinsulinemia in these rodent models do not accurately mimic the human condition.

### Insulin Resistance 

#### Mouse Models

In ob/ob mice, lack of leptin results in abnormally high levels of neuropeptide Y and increased cortisol levels, which underlie muscular insulin resistance [4]. Leptin administration inhibits insulin-stimulated glycogen synthesis in the muscle of ob/ob mice, and the basal levels of glycogen synthesis in untreated ob/ob mice are comparable to those of wild type control mice [108]. In contrast, in leptin-resistant human T2DM subjects, the insulin-stimulated glycogen synthesis was observed to be 50% lower than normal individuals [109]. Mitochondrial abnormalities are thought to be associated with insulin resistance as well. Mitochondrial genes involved in muscle mitochondrial respiration are up-regulated in human diabetes, but only a few of those enzymes are up-regulated in ob/ob mice [110].

Insulin receptor tyrosine kinase is directly involved in the cellular insulin signaling process [111]. T2DM patients have significant reduction in insulin-stimulated tyrosine kinase activity in the muscle as well as liver that underlies the development of insulin resistance [112, 113]. However, the activity of this enzyme in the muscle does not change in db/db mice [114]. Insulin resistance plays a central role in the pathogenesis of human T2DM, although it begins long before the onset of T2DM in the human population. In patients, almost all insulin resistance reflects defects in insulin-stimulated glucose transport into skeletal muscle cells. Fatty acids induce insulin resistance in skeletal muscle by the direct inhibition of insulin-activated glucose transport [113]. 

#### Rat Models

T2DM patients have decreased fat oxidative capacity [115], which may play a role in human muscle insulin resistance. However, in Zucker fatty rats, muscle fatty acid oxidation is typically increased, and different laboratories have reported conflicting results [116-118]. In addition, leptin-related hyperphagia is closely associated with the development of insulin resistance in this model [116-118], which may not be relevant to the situation in humans. Hepatic insulin receptor tyrosine kinase activity in SHR/N-cp rats does not contribute to the development of insulin resistance because it is not impaired, compared with control rats [119]. With regard to ZDF rats, although the receptor kinase is insensitive to insulin, the maximal insulin-stimulated activity is not altered, which is different from humans [120]. 

At 12 weeks of age, JCR:LA-cp rats are so insulin-resistant that there is no insulin-mediated glucose uptake by skeletal muscle [88]. The extreme insulin resistance observed in JCR:LA-cp rats is associated with significantly elevated muscle and tissue triglyceride observed as early as 4 weeks of age. It is also possibly secondary to hyperphagia and exacerbated by the absence of leptin-mediated inhibition of insulin secretion [88]. With regard to sex, male rats are more insulin resistant and hyperinsulinemic than female rats [63]. Leptin’s involvement in insulin resistance in these rodent models is complex and controversial. It appears that it contributes both to insulin-sensitization and insulin-resistance [121]. As a result, leptin signaling abnormalities in the models may complicate their insulin resistance, which does not represent the human T2DM condition. An important confounding factor is that even control rodents kept under standard laboratory conditions are “metabolically morbid” – they are “sedentary, obese, glucose intolerant, and on a trajectory to premature death” [122]. This can both influence additional levels of insulin resistance in T2DM rodent models and complicate interpretation and extrapolation of data to humans. Furthermore, “treatments shown to be efficacious in these animal models may prove ineffective or exhibit novel side effects in active, normal-weight subjects” [122]. 

### Macrovascular Complications

The morbidity and mortality of T2DM in humans primarily result from macrovascular and microvascular complications. Macrovascular complications include coronary artery disease, peripheral arterial disease and stroke. The key pathological mechanism underlying these conditions is atherosclerosis, resulting from hyperglycemia, chronic inflammation, and injury to the arterial wall [15]. 

#### Mouse Models

In general, mouse lipoprotein clearance is too efficient for atherosclerosis to develop spontaneously, and significant strain and sex variations exist [123]. Also, the reverse lipid profile (high HDL, low LDL) in ob/ob and db/db mice, compared with humans, leads to reduced atherogenic macrovascular disease risk compared with their nondiabetic controls [50], and a similar situation applies to Zucker rats and corpulent rats. The ob/ob and db/db mutations have been crossed onto atherosclerosis-prone strains of mice, but even in these models it is difficult to eliminate the confounding effects of leptin-related dyslipidemia from those of diabetes. Therefore, studies using ob/ob and db/db mice have focused on the early effects of obesity and insulin resistance on myocardial metabolism and function [123], rather than those on the vascular system, which is the major concern in human diabetic complications [15]. 

In ob/ob mice, leptin deficiency suppresses both innate and acquired immune responses [124]. In addition, leptin deficiency directly contributes to cardiac contractile dysfunction seen in this model, which is readily reversible with leptin replacement [125]. In contrast, leptin deficiency is clearly not a primary underlying cause of human diabetic cardiomyopathy. Unlike T2DM patients, these mice have reduced blood pressure, probably due to the loss of sympatho-excitatory actions of leptin [126]. In addition, hyperglycemia, the driving force of the development of diabetic complications in humans [15], is only transient in this model. As a result, the etiopathogenesis of macrovascular complications in ob/ob mice is different from that in human T2DM subjects. Db/db mice present cardiomyopathies, but like ob/ob mice, they also have similar or reduced systemic arterial blood pressure compared with lean controls [127-129]. In fact, the murine species as a whole have depressed heart rates and basal levels of systolic contraction compared to humans [130]. In the face of these conditions and the high levels of protective HDL cholesterol, atherosclerotic disease in db/db mice is reduced compared to the control heterozygous animals [50, 131]. 

#### Rat Models

Zucker fatty rats typically have moderate hypertension, although reports on this aspect are variable [52]. These rats do not develop premature atherosclerosis [57] or significant cardiovascular lesions [57], probably because they present no increase in LDL cholesterol even though they have decreased expression of hepatic receptors for LDL. As a result, they cannot be used as a model of human-like atherogenesis [132]. There are also sex differences in the mechanisms of increased serum cholesterol concentration [133]. In addition, vascular abnormalities are sometimes associated with chronic infection by mycoplasma, which is common in these rats [57]. Consequently, this model is not considered suitable to study macrovascular complications. ZDF rats do not develop hypertension or severe cardiovascular diseases [134-136]. It was reported that long-term severe diabetes in older ZDF rats induced only mild impairment of diastolic left ventricular function [136]. In contrast, clinically relevant diastolic dysfunction is common in human T2DM (usually as the first manifestation of cardiomyopathy), and it is correlated with duration and severity of diabetes [137]. 

SHR-cp rats develop minimal, if any, vascular or myocardial lesions, despite their hypertension [50]. Neither do they spontaneously develop atherosclerosis or large ischemic lesions [63]. With specific sub-strains or dietary manipulations, however, they can have fatal cardiomyopathy or fibrosis (personal communication with Dr. JC Russell). The JCR:LA-cp rat has been used as a model for diabetic atherosclerotic vascular disease, because unlike other rats, they spontaneously and rapidly develop myocardial lesions. However, such defects are only detected in males and the lesion occurrence decreases over the rats’ lifespan. Genetic drift has also been shown to reduce the cardiovascular events [50]. In addition, spontaneous myocardial infarctions also develop in response to stress, which is common in laboratory settings and confounds the diabetes-related complications. As a result, these rats must be handled gently with noise and lighting conditions tightly controlled [50]. Such manifestations clearly do not mimic human disease etiopathogenesis. Another caveat of this model is that platelet aggregation is not a critical component of the atherogenic processes in these rats as it is in human T2DM patients [50, 138]. These rats do not have hypertension compared with heterozygous control animals [62]. 

### Microvascular Complications

The pathological causes for T2DM microvascular complications are the same as for macrovascular complications, but the anatomical locations and manifestations differ. The hallmark human T2DM microvascular complications are retinopathy, nephropathy, and peripheral neuropathy. 

#### Mouse Models

ob/ob mice and Zucker fatty rats lack chronic hyperglycemia, a key underlying cause of microvascular complications in humans [15]. Consequently, these rodents are inaccurate models of these complications [139, 140]. Normal glucose levels may also contribute to the absence of diabetic nephropathy in ob/ob mice [75]. Microvascular diseases have been found in the retinal and renal vessels of db/db mice, and these mice are the most widely used model for mechanistic and interventional studies on diabetic nephropathy. Male db/db mice develop albuminuria at 10-12 weeks of age, and renal function declines at 15-18 weeks [141, 142]. While many research reports have come from this model, it also has limitations and does not fully reproduce the human conditions. For example, since db/db mice do not reliably have hypertension, the role of hypertension in the development of renal injury cannot be studied. This model also has less severe albuminuria and does not present significant glomerular basement membrane thickening [69]. In addition, renal interstitial fibrosis in db/db mice is mild; but it is one of the key features and a strong predictor of end-stage renal failure in diabetic nephropathy in T2DM human subjects [143]. Additionally, db/db mice do not develop mesangiolysis, nodular mesangial sclerosis, or progressive renal insufficiency which are features of advanced diabetic nephropathy [144]. Finally, since both human and db/db mouse kidneys mainly express leptin receptor Ob-Ra [145], and since the db/db mutation is in the Ob-Rb receptor, the leptin deregulation in this model may play a role in the pathogenesis of diabetic nephropathy that is not relevant to the conditions in humans. With regard to neuropathy, while db/db mice develop fiber atrophy [146] and profound neuropathy at 24 weeks after the onset of diabetes [147], they do not have increased sorbitol in the sciatic nerve [148], which is suggested to play an important role in the development of diabetic neuropathy in humans [149]. 

#### Rat Models

In Zucker fatty rats, the development of renal damage appears to be largely attributed to hyperlipidemia rather than hyperinsulinemia or diabetes, and the lipid profiles and clearance in rodents are not comparable to those of humans [75]. In addition to mild hyperglycemia, they have only borderline hypertension [150, 151], whereas hypertension significantly contributes to diabetic nephropathy in humans [152]. Although retinopathies were observed in ZDF rats, they are only present in diabetic male rats [153], and they do not have typical lesions of human diabetic retinopathy such as pericyte degeneration, microaneurysms, and acellular capillaries [153]. In addition, endothelium-dependent relaxation of intestinal microvasculature of ZDF rats is unimpaired, which is different from the substantially compromised conditions in humans [154]. The lack of hypertension in ZDF rats is also a drawback of this model in diabetic nephropathy. In addition, the lean littermates of diabetic ZDF rats also suffer renal lesions, which compromises the usefulness of this strain as a model for T2DM-associated nephropathy [134]. As for neuropathy, ZDF rats do not develop sympathetic neuroaxonal dystrophy, the hallmark of diabetic sympathetic autonomic neuropathy in humans [155]. 

The major functional complication in SHR/N-cp rats is renal dysfunction [87], although retinopathy and hearing loss were also observed and studied [156-158]. However, the contribution of hypertension to the pathogenesis of these conditions is complicated by the fact that although these rats were derived from a hypertensive rat strain, at 2 and 3.5 months of age the spontaneous hypertension is found in lean rather than obese male rats [86, 106], but at 8 months of age both lean and obese male rats are hypertensive to the same extent [158]. Also, renal vascular changes are rare in SHR/N-cp rats [159]. An early non-proliferative retinopathy is also observed in these rats, resulting from microangiopathy and electroretinographic deficits. But overall, the integrity of the retina is not compromised [158]. Lastly, neuropathy is not described in this strain of animals. 

Renal microvascular damages such as glomerulosclerosis are found in JCR:LA-cp rats [160], but other microvascular complications such as neuropathy and retinopathy are not described for this model. As with many other models described above, these rats are not hypertensive [62] and therefore do not completely represent the pathogenesis of microvascular complications in the majority of human T2DM patients. The microvascular complications of human T2DM usually develop relatively late in the disease process. Diabetic peripheral neuropathy takes years to decades to develop, and longer duration of diabetes increases the possibility of developing more than one form of neuropathy [161]. The short lifespan of rodents makes it difficult to study these complications. There is also a lack of uniformity in the diagnosis and monitoring of diabetic neuropathy in murine models due to substantial variability in the background strains, animal age and gender, and the type of diabetes present (including induction method and duration) [147].

### Other Features

#### Reproduction

The reproduction of each of these six rodent models is impaired, especially in those who are homozygous for leptin and leptin receptor mutations and thereby infertile [44, 53]. This is probably the direct result of the lack of leptin action associated with impaired hypothalamic-pituitary-gonadal feedback [54]. Whereas in human T2DM patients, fertility, especially related to leptin, does not constitute a major concern [162]. 

#### Leptin Expression and Signaling

Although obese T2DM patients are often leptin-resistant as well, there are differences in the regulation of leptin signaling between rodent models and humans. First, although the nucleotide sequences are highly homologous (~85%) in the coding region, there is only a 30% homology at 5′ and 3′ untranslated regions between mice and humans [24], indicating potentially substantial differences in the regulation of gene expression. As a comparison, the inter-individual DNA sequence variation in humans is only 0.1% to 0.15% [163]. With regard to leptin receptors, the amino acid sequences of mouse and rat Ob-Rb are 84% and 75% homologous to that of humans, respectively, while the homology is much higher between mice and rats (91%). Similar situations apply to other isoforms of leptin receptors as well [24, 28]. It is not uncommon that differences of just a few amino acid sequences can lead to drastic changes in the structures and/or functions of proteins. Indeed, a major difference between the human and mouse Ob-R is that the human receptor has a longer intracellular domain [164]. Additionally, there are 5 isoforms of Ob-R in mice, but only 3 in rats and 6 in humans [24, 28, 165]. 

In adult rodents, leptin mRNA levels are much higher in the gonadal and perirenal depots than in subcutaneous tissue [166]. In contrast, in adult humans, subcutaneous adipose tissue has higher levels of leptin mRNA [167, 168]. The differences in the distribution of leptin indicate corresponding differences in the regulation of leptin gene expression and signaling. Moreover, serum leptin levels in these models are usually much higher than those observed in humans and are prominently increased compared with control animals. In humans, leptin levels in obese T2DM patients are typically ~10 ng/ml, compared with ~5 ng/ml in non-diabetic obese subjects [169, 170]. The serum leptin level of db/db mice is much higher and quite variable, ranging from ~100 ng/ml in 4-week-old mice [171] to ~4000 ng/ml in 6-week-old mice [172]. Marked variability has also been shown in rats: Zucker fatty rats have shown serum leptin levels ranging from 0.15 ng/ml [173] to 10-50 ng/ml [174] up to 700 ng/ml [175] at overlapping ages. Serum leptin levels of male ZDF rats are 20-40 ng/ml and variable at ages 10-36 weeks [176-178]. In 5-month-old male SHR/N-cp rats, plasma leptin level is ~110 ng/ml [58]. Plasma leptin levels in male JCR:LA-cp rats increase from 40 to 80 ng/ml with increasing age from 4 weeks to 8 weeks, and at 4 weeks are already 30 fold higher than those in lean controls [179]. Due to leptin receptor deficiency in many of these models, the signaling pathways and cross-talk between insulin and leptin in these models are disrupted. Consequently, they do not mimic the adipoinsular axis of T2DM patients. 

#### Leptin/Leptin Receptor Transgenic Mice

In addition to rodents with spontaneously occurring leptin and leptin receptor mutations, several genetically modified lines with various phenotypes have been created. These models were generated primarily by knock-out/knock-in of leptin receptors (in a tissue-specific manner) and several key molecules involved in leptin signaling pathways. For example, mice with neuron-specific and hepatocyte-specific disruption of leptin receptor display various degrees of obesity syndrome and most are infertile [180, 181]. Collectively, these studies suggest that leptin exerts direct effects on neurons and that the majority of the weight-reducing effects of leptin are due to defective leptin signaling in the neurons and brain. In addition, transgenic mice overexpressing human leptin have yielded results not applicable to human T2DM [182-184]. Taken together, these studies shed little light on factors involved in human obesity, as leptin's effects on brain do not have the same influence on neuroendocrine or peripheral tissue effects associated with human obesity and T2DM. 

Binding of leptin to its cognate receptor initiates signal transduction cascades, and the most widely characterized cascade for leptin activation is the JAK/STAT pathway. Neuronal-specific deletion of key signaling molecules such as STAT3, SHP2, and Foxo1 in this pathway leads to varying degrees of leptin resistance, obesity syndrome, and fertility issues [185]. Similarly, these studies are of limited use, if any, for characterizing signaling cascades associated with human obesity since deficiencies in leptin signaling cascades do not significantly influence human obesity. Furthermore, deleting molecules commonly shared by many crucial signaling pathways, especially insulin signaling [186], could have potentially deleterious effects on overall cellular signaling cascades, which makes it difficult to delineate those factors that specifically contribute to obesity and T2DM. In addition, studies have been done in leptin- and leptin receptor-deficient animals in order to discover potential genes of interest in human T2DM by examining the strain differences that lead to different severities of diabetic manifestations. For example, the genes *tomosyn-2* (syntaxin-binding protein 5-like) and *LI *(Lisch-like) were identified by this approach [187-190]. As most of these studies are in their early stages, the relevance of these discoveries to human T2DM remains to be understood.

## CONCLUSION AND PERSPECTIVES

Although leptin- and leptin receptor-deficient animals present obesity and some T2DM-like manifestations, these manifestations are in fact secondary to genetic mutations that do not reflect disease etiology in their human counterparts. One of the most striking and distinct features of these rodent T2DM approximations is their monogenic inheritance pattern. In addition, these animals have been inbred for many generations and their genetics are homogeneous. This is in contrast to the etiology of human T2DM, which is not only polygenic, but also multifactorial in nature, on a non-homogenous genetic background. Furthermore, these heterogeneous human populations also reflect important contributions from acquired factors. As a result, the information obtained from these animal models is of limited use for understanding the etiology of the much more complex human T2DM. 

The scientific rationale for studying T2DM-like manifestations in leptin- and leptin-receptor deficient rodents is also flawed since leptin or leptin receptor deficiency is not an important contributor to T2DM in humans. Furthermore, both the abnormal leptin signaling and the related systemic effects beyond appetite control and energy metabolism in these models confound translation. Human T2DM complications develop over many years and display characteristic pathophysiological hallmarks, in contrast to leptin- and leptin receptor-deficient rodent models of T2DM. In addition, the presence or absence of certain diabetic manifestations is often dependent on the diet, sex, and age of these animals, often making it difficult to compare rodent data both within and between laboratories, let alone translate findings to human T2DM. All of these issues are further confounded and modulated by strain-dependent variability that exists within the Mus and Rattus genera. 

In addition to the genetic basis of T2DM, rodent models of T2DM in general significantly differ from human T2DM at every level of glucose regulation from nucleic acid to the maintenance of whole-body glucose homeostasis, extending to the population level [191]. These insuperable species differences significantly impair the ability to reliably extrapolate across species. While the animal models described here display some pathways and manifestations similar to human T2DM, the underlying mechanisms and the regulation of these pathways may be very different, and many details remain unknown even after decades of rodent research. A common rationale for *in vivo* animal studies is that whole animal models presumably mimic the complex intercellular and inter-systemic crosstalk in humans. However, considering the many known and unknown differences between these models and humans, findings in these animals can be challenging to interpret and very difficult to reliably extrapolate to humans. There is no clear solution to overcome the translational gap between these animal models and humans, particularly since the complex genetic determinants are likely immutable. Alternative strategies directly applicable and relevant to humans should be utilized and further developed to overcome the translational barrier – it appears that a shift from leptin and leptin receptor-deficient rodent models to human-based T2DM research methods will facilitate directly human-relevant information that will enable scientists to effectively battle against the current obesity and diabetes pandemics.

## Figures and Tables

**Table 1.** Comparison of diabetic manifestations in leptin- and leptin receptor-deficient models and human T2DM.

	ob/ob Mouse		db/db Mouse	Zucker Fatty Rat	ZDF Rat	SHR/N-cp Rat	JCR/LA-cp Rat	Human T2DM Patients
**Obesity**	Severe, early onset		Severe, early onset	Severe, early onset	Severe, early onset	Severe, early onset	Severe, early onset	Moderate, variable onset
	Obesity largely due to hyperphagia caused by leptin signaling deficiency							Multifactorial causes
**Dyslipidemia**	Hyperlipidemia contributed to by high levels of HDL			Hyperlipidemia due to high LDL and HDL, and LPL activity is increased	Hyperlipidemia due to high LDL and HDL, and LPL activity is increased	Hyperlipidemia due to high LDL and HDL	Hyperlipidemia mainly due to high VLDL, LPL activity is increased	Dyslipidemia often characterized by reduced HDL, elevated LDL and VLDL, and decreased LPL activity
**Hyperglycemia**	Mild and transient		Severe, but not all animals become hyperglycemic	Normal or mild, partially due to sturdy pancreas	Severe in males, but normal in females	Post-prandial hyperglycemia, normal fasting glucose	Moderate in female, normal fasting glucose	Moderate in both genders, with men more susceptible than women
**Hyperinsulinemia**	Severe throughout life		Severe from early in life	Severe early in life, back to normal at old age	Severe from early in life	Severe from early in life	Severe from early in life	Moderate, before the onset of diabetes later in life
**Pancreatic β-cell dysfunction**	No		Yes	No	Yes	No	No	Yes
**Pancreas pathology**	No islet amyloid deposition							Amyloidosis
**Insulin resistance**	Yes		Yes	Yes	Yes	Yes	Yes	Yes
**Macrovascular complications**	No spontaneous atherosclerosis						Spontaneously atherosclerotic	Atherosclerosis is the key underlying cause of diabetic complications, hypertension and chronic hyperglycemia also play important roles in the pathogenesis of these complications
	Reduced systemic arterial blood pressure, substantially depressed heart rates and basal systolic contraction than humans			Borderline hypertension, no significant cardiovascular lesions	No hypertension, mild cardiac dysfunction	Essential hypertension, but not in obese male rats. Minimal if any spontaneous vascular or myocardial lesions	Normotensive, spontaneous myocardial lesions	
**Microvascular complications**	Life span too short to simulate human conditions							
	Lack of hypertension, hyperglycemia and/or atherogenesis compromises their usefulness							
	A prediabetic model with mild or no renal disease	Nephropathy lacking features of advanced condition		A prediabetic model	Nephropathy confounded by nondiabetic lesions; Retinopathy and retinopathy lacking typical lesions in humans	Nephropathy, but renal vascular changes are rare. Has retinopathy and hearing loss	Nephropathy	Nephropathy, neuropathy and retinopathy
